# Understanding the impact of the COVID-19 pandemic response on GI infection surveillance trends in England, January 2020–April 2022

**DOI:** 10.1017/S095026882300136X

**Published:** 2023-08-25

**Authors:** Nicola K. Love, Amy Douglas, Saheer Gharbia, Helen Hughes, Roger Morbey, Isabel Oliver, Gillian E. Smith, Alex J. Elliot

**Affiliations:** 1 North East Field Services, Health Protection Operations, UK Health Security Agency, Newcastle upon Tyne, UK; 2 National Institute for Health Research Health Protection Research Unit (NIHR HPRU) in Gastrointestinal Infections, University of Liverpool, Liverpool, UK; 3 Gastrointestinal Infections and Food Safety (One Health) Division, UK Health Security Agency, London, UK; 4 Real-time Syndromic Surveillance Team, Field Service, Health Protection Operations, UK Health Security Agency, Birmingham, UK; 5 Farr Institute@HeRC, University of Liverpool, Liverpool, UK; 6 National Institute for Health Research Health Protection Research Unit (NIHR HPRU) in Emergency Preparedness and Response, King’s College London, London, UK; 7 Science Group, UK Health Security Agency, London, UK; 8 National Institute for Health Research Health Protection Research Unit (NIHR HPRU) in Behavioural Science and Evaluation, Population Health Sciences, University of Bristol, Bristol, UK

**Keywords:** COVID-19, foodborne infections, gastrointestinal infections, non-pharmaceutical interventions, surveillance

## Abstract

Stepwise non-pharmaceutical interventions and health system changes implemented as part of the COVID-19 response have had implications on the incidence, diagnosis, and reporting of other communicable diseases. Here, we established the impact of the COVID-19 outbreak response on gastrointestinal (GI) infection trends using routinely collected surveillance data from six national English laboratory, outbreak, and syndromic surveillance systems using key dates of governmental policy to assign phases for comparison between pandemic and historic data. Following decreases across all indicators during the first lockdown (March–May 2020), bacterial and parasitic pathogens associated with foodborne or environmental transmission routes recovered rapidly between June and September 2020, while those associated with travel and/or person-to-person transmission remained lower than expected for 2021. High out-of-season norovirus activity was observed with the easing of lockdown measures between June and October 2021, with this trend reflected in laboratory and outbreak systems and syndromic surveillance indicators. Above expected increases in emergency department (ED) attendances may have reflected changes in health-seeking behaviour and provision. Differential reductions across specific GI pathogens are indicative of the underlying routes of transmission. These results provide further insight into the drivers for transmission, which can help inform control measures for GI infections.

## Introduction

As a result of the COVID-19 pandemic, governments implemented stepwise non-pharmaceutical intervention (NPI) measures designed to mitigate the impacts of the virus and reduce transmission, while undertaking changes to healthcare provision and patient management to reduce the strain on health systems. Many of the measures introduced such as improved hand hygiene, reduced social contact, increased environmental cleaning, and the closure of premises are control measures effective in reducing the incidence of other communicable diseases [1].

Gastrointestinal (GI) infections are an important cause of morbidity and mortality globally, placing a considerable burden on primary and secondary healthcare services [2]. We previously reported on marked changes in the trends of GI infections during the initial 6 months of the COVID-19 outbreak response in England (the first partial lockdown and relaxation of initial lockdown measures; [3]). This initial work demonstrated a 52% decrease in GI outbreaks and a 34% decrease in laboratory-confirmed cases reported during the first national lockdown with figures remaining lower than historic averages once measures were eased. Similar rapid reductions in gastroenteric pathogens were consistently observed across surveillance systems in other countries with the onset of the pandemic in 2020, with decreases estimated to be around 70–80% for reported viral GI outbreaks [4, 5] and between 19 and 55% for laboratory-confirmed bacterial GI pathogens [6–10]. Several hypotheses were proposed to explain the reductions in GI pathogens observed. These included changes in health-seeking behaviour, limited testing, changes in healthcare provision, and true decreases in incidence. Following the lifting of all control measures in England on 24 February 2022, we now present an update to our initial study, which aims to establish the impact of subsequent pandemic lockdowns, control measures, and easing of measures on GI infection surveillance trends over the pandemic response in England.

## Methods

A retrospective ecological study was conducted by performing secondary analyses on routinely collected national surveillance data from six national UK Health Security Agency (UKHSA) coordinated systems, as described previously [8]. Systems included outbreak monitoring (HPZone), laboratory notifications from all National Health Service (NHS) laboratories (Second Generation Surveillance System; SGSS), and syndromic surveillance systems (NHS 111 telehealth consultations, general practitioner (GP) ‘in-hours’ and ‘out-of-hours’ consultations, and emergency department (ED) attendances [11, 12]).

**Figure 1.**
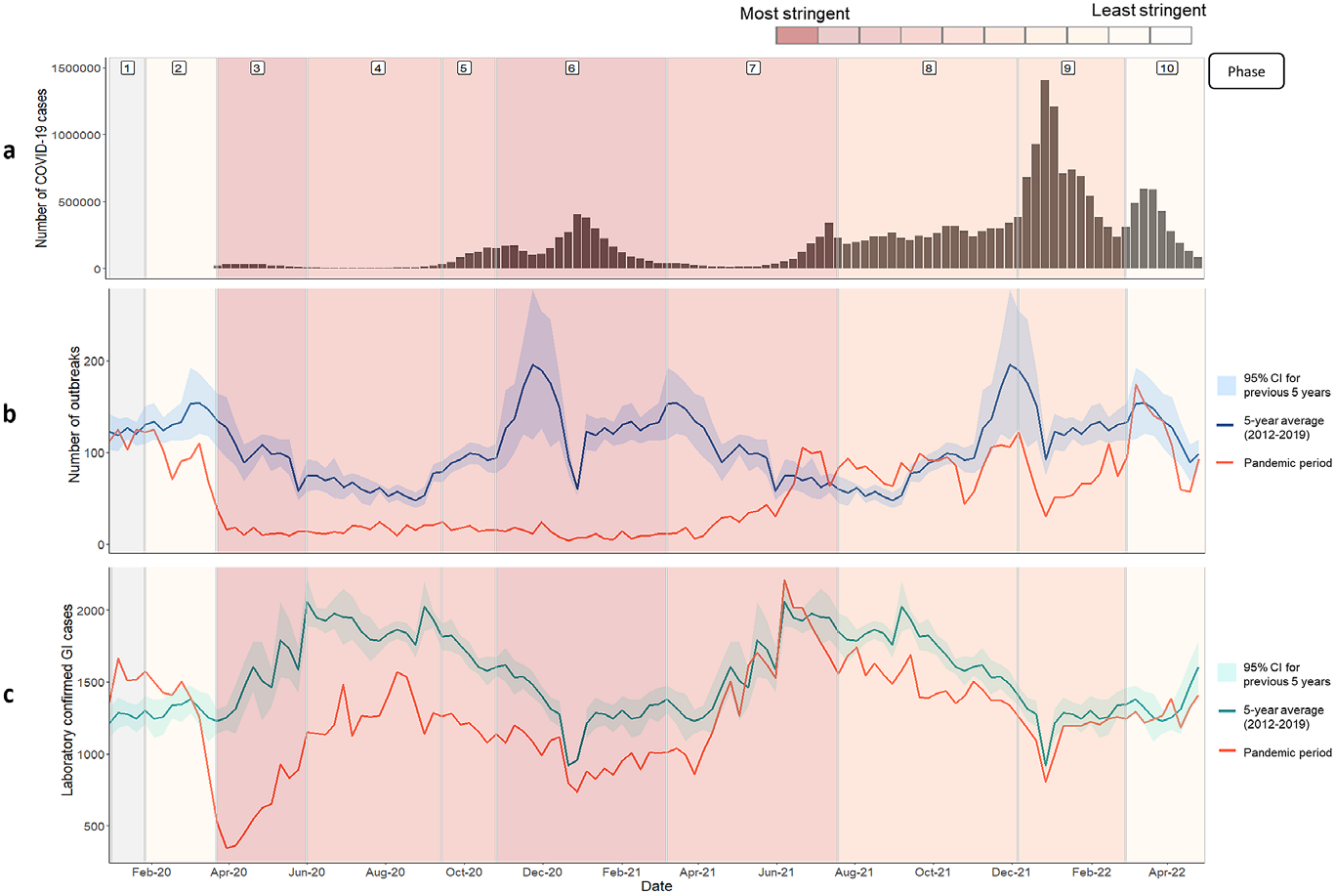
Data covering the period between January 2020 and May 2021 split into 10 pandemic phases showing A) Laboratory confirmed COVID-19 cases in England reported via the UK COVID-19 dashboard (https://coronavirus.data.gov.uk/) (B) Gastrointestinal outbreaks reported to the UK Health Security Agency (UKHSA) and entered into HPZone by week of date recorded during the pandemic period (red line) and 5-year historic average and associated 95% confidence interval (blue line) and C) Laboratory confirmed gastrointestinal infections* reported to the UKHSA by specimen date during the pandemic period (red line) and 5-year historic average and associated 95% confidence interval (green line). Pandemic phases are assigned based on control measures implemented during the pandemic response, using the Oxford Stringency Index which indicates the severity of government restrictions in England [13] from least severe measures to most severe measures. A weekly stringency index was calculated based on the mean score of nine metrics: school closures; workplace closures; cancellation of public events; restrictions on public gatherings; closures of public transport; stay-at-home requirements; public information campaigns; restrictions on internal movements; and international travel control, each taking a value between 0 and 100, with 100 being the strictest response.This score was converted to deciles, as displayed in the bar at the top of the figure. Grey shaded area indicates no restriction measures in place during early January 2020. * Laboratory confirmed gastrointestinal infections (Campylobacter spp., Cryptosporidium spp., Shiga-toxin producing E. coli [STEC], Giardia sp., Norovirus, non-typhoidal Salmonella spp., Shigellaspp), reported by NHS laboratories to UKHSA’s SGSS laboratory surveillance system.

For laboratory and outbreak datasets, the COVID-19 pandemic period was defined as 30 December 2019 to 30 April 2022 inclusive, with historic comparator data covering 29 December 2014 to 29 December 2019 inclusive aggregated by ISO week. For syndromic surveillance indicators, the historic comparator period covered 31 December 2018 to 29 December 2019 inclusive. Data were further split into COVID-19 pandemic ‘phases’ for comparison, determined by the stringency of control measures using the Oxford Severity Scale [13] and key dates of UK governmental policy ([14]; Figure 1). These phases were defined as follows: ‘phase 1’, the pre-outbreak period; ‘phase 2’, advising of hygiene and social distancing measures prior to the first national lockdown; ‘phase 3’, the first national lockdown; ‘phase 4’, the gradual easing of restriction measures; ‘phase 5’, the reimplementation of restriction measures; ‘phase 6’, the second and third national lockdowns; ‘phase 7’, the easing of restriction measures; ‘phase 8’, the removal of legal limits on social contact and reopening of all closed sectors; ‘phase 9’, the reintroduction of ‘Plan B’ measures to limit the spread of the emerging Omicron variant in December 2021; and ‘phase 10’, the removal of all restrictions as part of the UK’s ‘Living with COVID-19’ strategy ([14]; Supplementary Table S1). Phases were based on nationwide policy and did not account for regional or local differences in restriction measures implemented as part of England’s tier system [14].

Cumulative weekly outbreaks recorded in HPZone and pseudonymised SGSS data for selected laboratory-confirmed organisms (*Campylobacter* spp., *Cryptosporidium* spp., Shiga toxin-producing *Escherichia coli* (*E. coli*) [STEC], *Giardia* sp., *Listeria* spp., Norovirus, non-typhoidal *Salmonella* spp., and *Shigella* spp.) were grouped by a week of notification date (HPZone) or specimen date (SGSS).

Outbreak and laboratory data from the pandemic period were plotted in a time series together with the weekly 5-year average and 95% confidence intervals (2015–2019) and superimposed COVID-19 outbreak phases, unless otherwise specified. Syndromic data were analysed as described elsewhere [15]. Daily incidence rates per 100,000 attendances or patient contacts were calculated using a denominator of number of people using or contacting each service each day for all health conditions and a numerator of diarrhoea and/or vomiting or gastroenteritis, with 7-day moving averages determined. The same period in 2019 was used as a comparator for 2020 to 2022. Established syndromic surveillance data were complemented by Google Trends data, as web searches are unlikely to be as impacted as healthcare services by capacity issues [8]. Google Trends searches were performed for key phrases associated with GI illness in England, as described previously, with a score of 100, which is used to represent relative search interest over the given time period and geography. Analyses were in RStudio, PBC, Boston, MA (version 2022.7.1.554).

Ethical approval was not required for this study as data collection is conducted as part of the routine surveillance of communicable diseases under the provisions of Section 251 of the NHS Act 2006 and therefore does not require individual patient consent. The study was reviewed by the UKHSA Research Ethics and Governance Group and was found to be fully compliant with all regulatory requirements.

## Results

### Outbreak activity

GI outbreak activity decreased during the first lockdown with activity remaining significantly lower than historic figures despite the easing of measures from June 2020 (phase 4; Figure 1b). Indeed, outbreak activity remained below expected for 2020 and into the 2020/21 winter season where a 92% reduction in reported outbreaks was observed (November 2020–March 2021; phase 6; 208 versus 2,484 outbreaks (95% CI: 1,902–3,106)). Low outbreak activity over the 2020/21 winter season coincided with England’s second and third national lockdowns during phase 6 (31 October–2 December and 4 January 2021–8 March 2021). GI outbreaks reported in England are predominantly viral in nature occurring within health and social care settings, particularly from October to April [3].

Outbreak activity began to gradually increase from March 2021 as lockdown restriction measures were gradually lifted (phase 7), returning to normal limits or higher between June and October 2021 (weeks 24–43; phases 7 and 8), before decreasing with the implementation of ‘Plan B’ measures in response to the emergence of the new Omicron variant in December 2021 (December 2021–February 2022; phase 9; 48% reduction; 844 versus 1,614 outbreaks (95% CI: 1,294–1,935)). With the removal of all restriction measures as part of the UK Government’s ‘Living with COVID’ strategy, outbreak activity increased to the highest level seen during the study period, though remained within historic limits in phase 10 (March–April 2022).

Reductions in suspected and confirmed viral, protozoal, and bacterial outbreaks were observed across the pandemic period (phases 2–9), with a 58% reduction in the number of reported suspected and confirmed viral outbreaks (3,946 versus 9,376 outbreaks), a 90% reduction in protozoal outbreaks (14 versus 142), and a 41% reduction in bacterial outbreaks (244 versus 414 outbreaks; 41% decrease) reported.

### Laboratory surveillance

Following initial decreases, cases of laboratory-confirmed GI pathogens began to increase during the first lockdown (phase 3; Figure 1c) but remained significantly lower than historic values for 2020 (68,394 versus 82,063 cases (95% CI: 77,437–86,689)), although small peaks in activity were observed in phase 4 (July–September 2020). During the second and third UK lockdowns (phase 6; November 2020–March 2021), laboratory-confirmed cases did not decrease to the same extent as the first lockdown, although activity remained lower than historically observed (23% decrease; 18,726 versus 24,407 (95% CI: 23,243–25,570)). Cases returned to just under expected limits with the easing of restriction measures in March 2021 (phase 7) and remained 15% below historic figures until January 2022 when activity returned to within normal limits.

**Figure 2.**
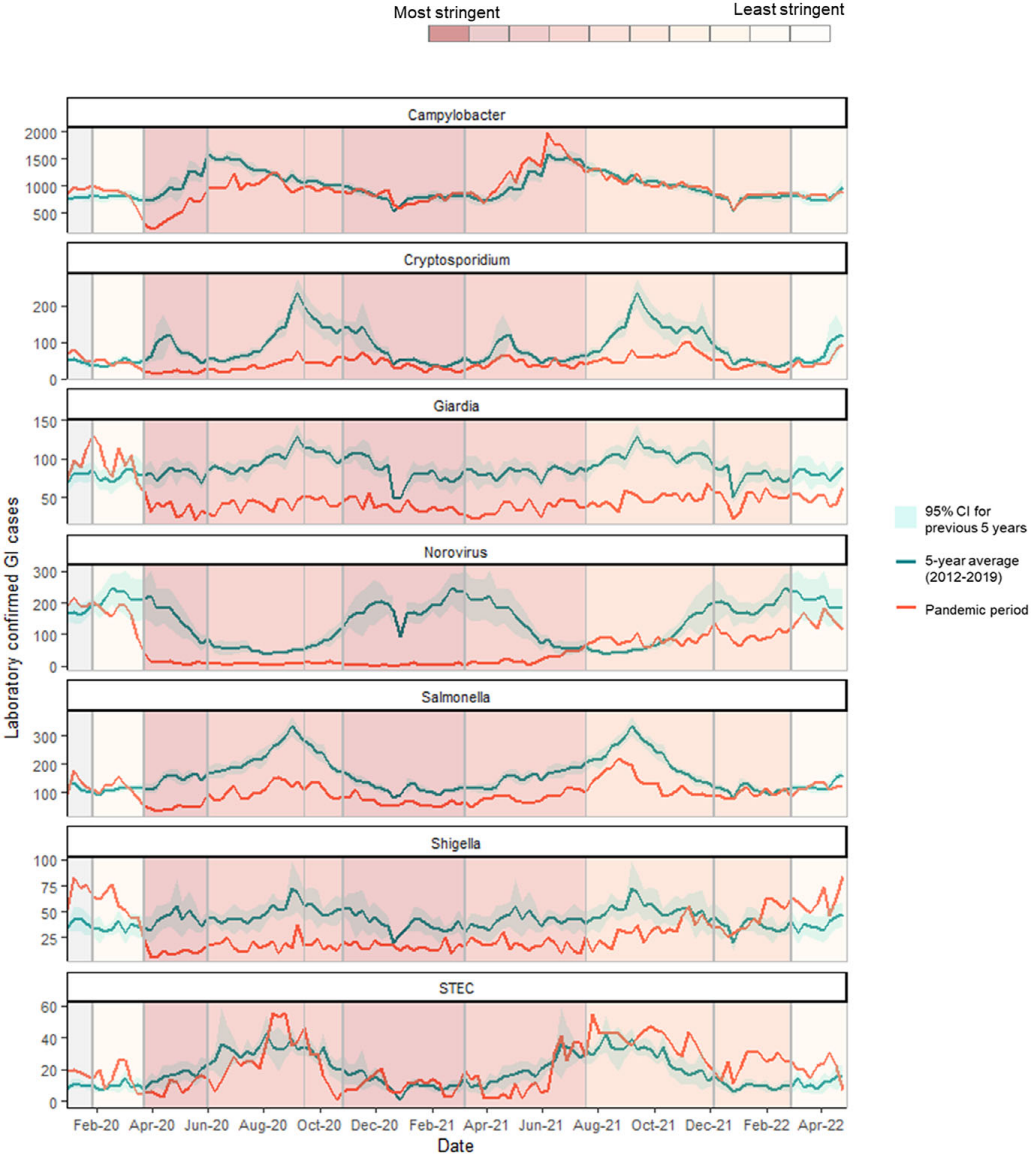
Data covering the period between January 2020 and May 2021 split into 10 pandemic phases showing laboratory confirmed gastrointestinal pathogens* reported to UKHSA by specimen date during the pandemic period (red line) and 5-year historic average and associated 95% confidence interval (green line) for Campylobacter, Cryptosporidium, Giardia, Norovirus, Salmonella, Shigella and STEC**. Pandemic phases are assigned based on control measures implemented during the pandemic response, using the Oxford Stringency Index which indicates the severity of government restrictions in England [13] from least severe measures to most severe measures. A weekly stringency index was calculated based on the mean score of nine metrics: school closures; workplace closures; cancellation of public events; restrictions on public gatherings; closures of public transport; stay-at-home requirements; public information campaigns; restrictions on internal movements; and international travel control, each taking a value between 0 and 100, with 100 being the strictest response. This score was converted to deciles, as displayed in the bar at the top of the figure. Grey shaded area indicates no restriction measures inplace. *Laboratory confirmed gastrointestinal infections (Campylobacter spp., Cryptosporidium spp., Shiga-toxin producing E. coli [STEC], Giardia sp., Norovirus, non-typhoidal Salmonella spp., Shigella spp), reported by NHS laboratories to UKHSA’s SGSS laboratory surveillance system ** Listeria is not included in Figure 2 due to suppression of small numbers.

Cumulative GI trends are strongly influenced by Campylobacter activity, the most common pathogen diagnosed by diagnostic laboratories in England. Therefore, we determined the trend for each GI pathogen separately across the pandemic period (phases 2–9; Figure 2). Listeria (not shown in Figure 2 due to small numbers) and STEC activity quickly returned to expected levels from May 2020 with the easing of lockdown measures, while Campylobacter diagnoses returned to normal in November 2020 (phase 6). Diagnoses of these pathogens then remained comparable to or higher than historic figures for the remainder of the pandemic period (STEC: 9% increase over the pandemic period; 2,366 versus 2,175 cases (95% CI: 1,514–2,823 cases); Listeria spp.: 15% increase: 379 versus 330 (95% CI: 145–514); and *Campylobacter* spp.: 6% decrease: 104,668 versus 111,452 (95% CI: 100,592–121,050)). In comparison, reports of laboratory-confirmed Cryptosporidium (52% decrease: 4,629 versus 9,547 (95% CI: 7,501–11,482)), Giardia (48% decrease: 5,046 versus 9,769 (95% CI: 8,440–11,003)), Shigella (41% decrease: 2,810 versus 4,781 (95% CI: 3,442–6,064)), and Salmonella (42% decrease: 10,644 versus 18,353 (95% CI: 16,337–20,188)) remained substantially lower than historic figures across the pandemic period, with activity for all bacterial and parasitic pathogens with the exception of giardia returning to within or above expected figures in early 2022.

Norovirus activity remained lower than expected for 2020 and into the 2020/21 winter season. Activity then increased in May 2021 (phase 7), corresponding with the easing of restriction measures and reopening of schools and nurseries. Unusually, high norovirus activity continued throughout the summer of 2021 exceeding the 5-year average over a 2-month period in August and September before plateauing in the winter of 2021/22, which coincided with the emergence of the Omicron COVID-19 variant. Across the pandemic period, cases were lower than expected based on historic figures (66% decrease: 5,030 versus 14,798 cases (95% CI: 10,843–18,567)).

### Syndromic surveillance

**Figure 3.**
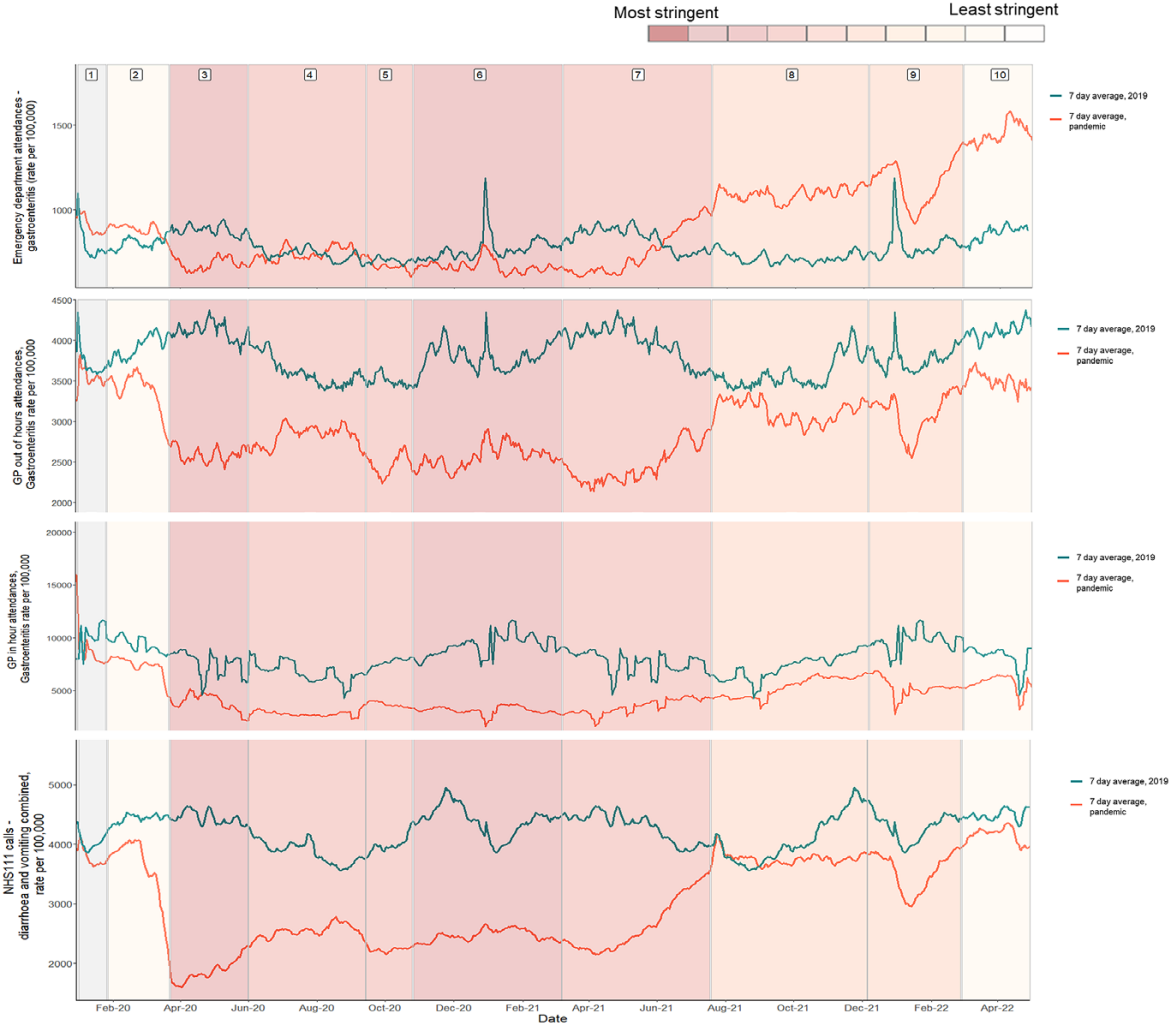
Data split into 10 pandemic phases showing A) emergency department attendances B) GP out of hours contacts C) GP in hours consultations and D) NHS111 calls. For emergency department, GP out of hours and GP in hours indicators show both the pandemic period and historic 2019 comparator, with the rolling 7-day average rate per 100,000 attendances for the pandemic period indicated in red and the historic comparator in blue. NHS111 calls show combined calls for diarrhoea and vomiting with the rolling 7-day average rate per 100,000 calls for the pandemic period indicated in red and the historic 2019 comparator in green. Pandemic phases are assigned based on control measures implemented during the pandemic response, using the Oxford Stringency Index which indicates the severity of government restrictions in England [13] from least severe measures to most severe measures. A weekly stringency index was calculated based on the mean score of nine metrics: school closures; workplace closures; cancellation of public events; restrictions on public gatherings; closures of public transport; stay-at-home requirements; public information campaigns; restrictions on internal movements; and international travel control, each taking a value between 0 and 100, with 100 being the strictest response. This score was converted to deciles, as displayed in the bar at the top of the figure. Grey shaded area indicates no restriction measures in place. Pandemic phases are assigned based on control measures implemented during the pandemic response, using the Oxford Stringency Index which indicates the severity of government restrictions in England [13] from least severe measures to most severe measures. A weekly stringency index was calculated based on the mean score of nine metrics: school closures; workplace closures; ancellation of public events; restrictions on public gatherings; closures of public transport; stay-at-home requirements; public information campaigns; restrictions on internal movements; and international travel control, each taking a value between 0 and 100, with 100 being the strictest response. This score was converted to deciles, as displayed in the bar at the top of the figure. Grey shaded area indicates no restriction measures in place.

**Figure 4.**
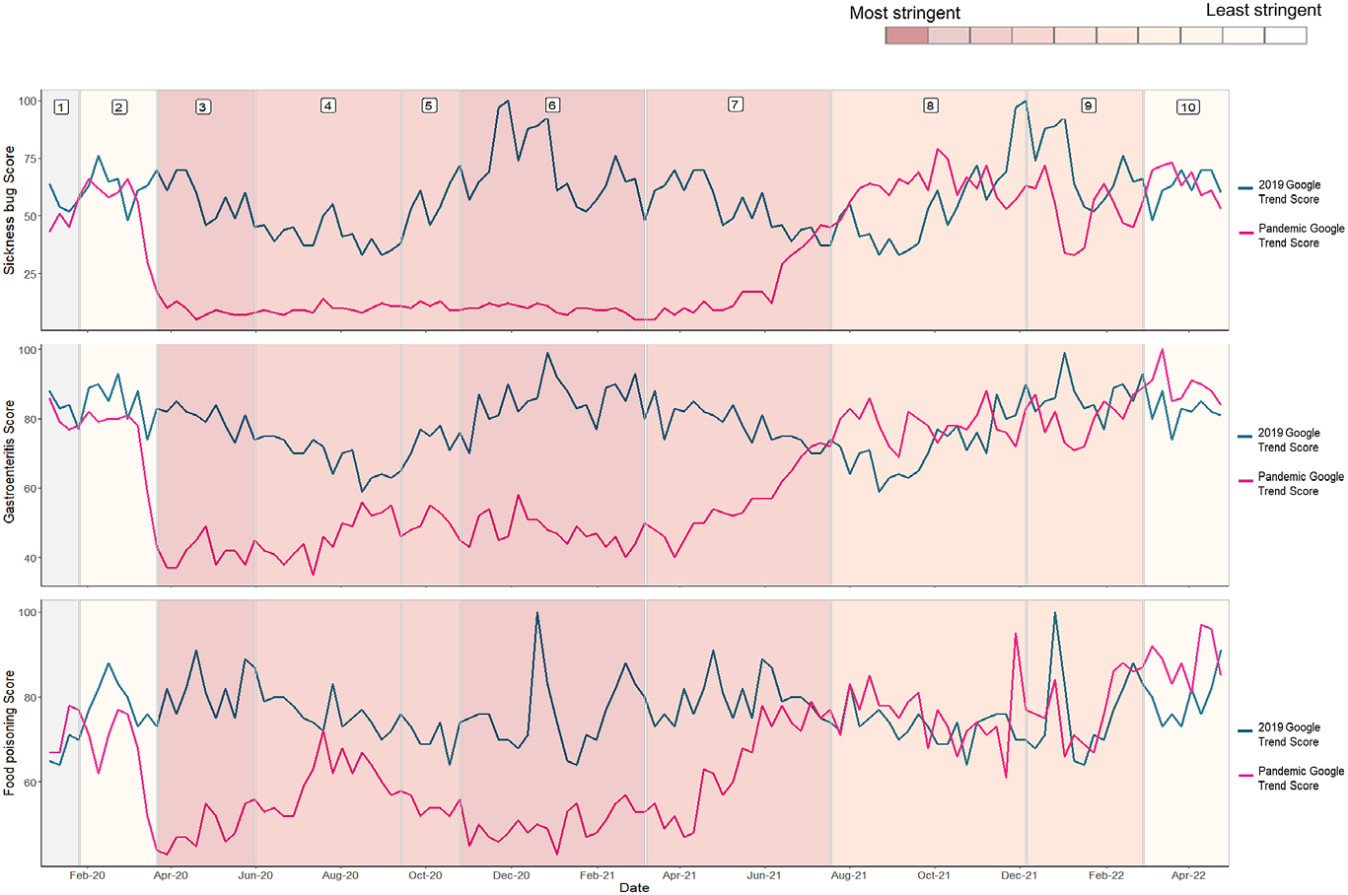
Relative search volume for the Google search terms A) Sickness bug, B) Gastroenteritis and C) Food poisoning determined using Google Trend data restricted to England for the pandemic period (red line) and 2019 (green line).Pandemic phases are assigned based on control measures implemented during the pandemic response, using the Oxford Stringency Index which indicates the severity of government restrictions in England [13] from least severe measures to most severe measures. A weekly stringency index was calculated based on the mean score of nine metrics: school closures; workplace closures; cancellation of public events; restrictions on public gatherings; closures of public transport; stay-at-home requirements; public information campaigns; restrictions on internal movements; and international travel control, each taking a value between 0 and 100, with 100 being the strictest response. This score was converted to deciles, as displayed in the bar at the top of the figure. Grey shaded area indicates no restriction measures in place.

Syndromic surveillance data were used to determine healthcare utilisation for gastroenteritis or diarrhoea and vomiting combined across several syndromic indicators. Following initial decreases across all indicators during the first lockdown, emergency department (ED) attendances returned to 2019 levels during the easing of lockdown measures in both 2020 (phase 4) and 2021 (phase 7) but remained below historic figures across the 2020/21 winter season (Figure 3a). Interestingly, while GP out-of-hours (Figure 3b) and GP in-hours (Figure 3c) consultations remained lower than in 2019, despite small increases in July 2021, which coincided with the easing of measures, there were increases in ED attendances above what would be expected based on 2019 data. NHS 111 calls for diarrhoea and vomiting showed a similar trend to other syndromic systems (Figure 3d), with a decrease in activity before the first lockdown, a low number of calls across the pandemic period, and an increase in activity with the easing of measures in mid-2021. Established syndromic surveillance data were complemented by Google Trends data, with searches for the terms ‘Sickness bug’, ‘Gastroenteritis’, and ‘Food poisoning’ again showing low activity across the pandemic period until the easing of restriction measures in mid-2021 (Figure 4).

## Discussion

There is increasing evidence that NPIs and health system changes introduced during the COVID-19 pandemic in early 2020 led to reductions in communicable infections, including enteric pathogens [4-10]. The drivers of change were hypothesised to be multifactorial; changes to health-seeking behaviour, pressure on diagnostic services, and surveillance system ascertainment have undoubtedly all played a role; however, there has likely been a true decrease in the incidence of infections resulting from the control measures and restrictions implemented. Here, through the triangulation of several national surveillance systems, we provide further evidence to support the hypothesis of a true decrease in the incidence of certain GI pathogens by describing GI infection trends in England during the entirety of COVID-19 NPIs and restrictions up to the removal of all restrictions in April 2022.

We show that following the end of phase 3, the first lockdown in 2020, the activity of foodborne pathogens such as STEC and Campylobacter quickly returned to historic levels, while pathogens more associated with person-to-person transmission or foreign travel (and therefore more influenced by hand hygiene, social distancing measures, and travel regulations) recovered at a slower rate [1]. These findings suggest that although rapid changes to healthcare provision altered health-seeking behaviour and non-COVID-19 testing capacity likely resulted in initial decreases observed across surveillance systems [3], there were true pathogen-specific reductions in incidence as a result of implemented measures.

Across the pandemic period, the activity of enteric pathogens correlated with stages in the COVID-19 response. Periods of higher pathogen activity corresponded to the relaxation of initial NPIs and lower activity identified with the reimplementation of further control measures with subsequent COVID-19 waves. This was particularly striking for norovirus, where the reimplementation of further measures in the 2020/21 and 2021/22 winter seasons was followed by substantially lower laboratory-confirmed and outbreak activity than expected, while increased out-of-season activity followed the easing of measures in the summer of 2021.

Increases in GI activity following the relaxation of NPIs were also observed in surveillance data from other countries. As in England, unseasonal activity of norovirus occurred in Germany in the summer and Autumn of 2021, following the relaxation of measures [16-17], with the same trend also observed for several respiratory viruses [16]. Furthermore, data from Australia showed that the removal of NPIs coincided with an increase in gastroenteritis outbreaks in childcare settings [4]. Reductions in viral GI pathogen activity also coincided with decreases in outbreak number and average outbreak size [10] and reductions in both the number of enteric samples submitted and the proportion of positive samples [5]. In the UK, most reported viral outbreaks are linked to health and social care settings [3]; therefore, some of the reduction observed is likely due to restricted access to and restricted movement within such settings in addition to enhanced hygiene measures.

The differing trends observed for a diverse range of enteric pathogens provide support to the hypothesis of a true decrease in incidence with differences between pathogens also observed in laboratory surveillance systems in Korea, China, Germany, and Poland [6, 7, 18-20]. These studies showed that bacterial pathogens were non-significantly different from historic figures over the 2020 period, but viral pathogens were significantly reduced. The differences observed are likely due to the varied modes of transmission between pathogens. Viral pathogens are more likely to be transmitted from person to person, while bacterial pathogens are more commonly transmitted via foodborne routes. The setting of ‘acquisition of infection’ also differs between viral and bacterial pathogens, with viral pathogens frequently associated with closed settings such as schools, care homes, and hospitals or with food premises, while bacterial pathogens are often acquired from food products consumed within the home. An earlier study from England found a stronger association between norovirus activity and changes in the stringency of COVID-19 control measures than that for Campylobacter [21]. Foreign travel is an important risk factor for many bacterial pathogens in England [1,3]. Here, we show that while diagnoses of certain bacterial pathogens returned to normal in England, as in other countries, other pathogens such as Salmonella remained lower than historic figures for 2020/2021. This was comparable to findings from the USA where both bacterial and viral gastrointestinal pathogens were reduced [18] and likely reflect the impact that restrictions on non-essential foreign travel have had on organisms for which foreign travel is an important risk factor [1,3].

While there may be a true difference in the incidence of bacterial and viral pathogens resulting from differences in transmission mechanism, it is also possible that changes in healthcare-seeking behaviour, differing presentations, and the management of laboratory testing for pathogens may have contributed to the changing trends observed. It has previously been suggested that changes observed in laboratory reporting surveillance data in 2020 were due to underreporting because of reduced testing capacity [21, 22]; however, core national laboratory capacity was not used to support mass public SARS-CoV-2 testing from May 2020, which will have minimised the impact on laboratory capacity for non-COVID-19 testing during the remainder of the pandemic [23]. Further consideration must be made of the reduced capacity available to the wider public health system and changes to healthcare-seeking behaviour resulting in fewer samples being submitted for testing. It is also important to note that changes to laboratory processing and ascertainment cannot explain the impacts observed across non-laboratory systems such as syndromic surveillance systems. Although healthcare systems used for syndromic surveillance may be impacted by reduced availability of healthcare services, Google Trends web search data are unlikely to be impacted by such capacity issues and showed a similar trend to more formal syndromic surveillance systems. It is also possible that individuals with more severe and prolonged infections such as STEC, which tend to be bacterial, were more likely to access care or that bloody diarrhoea specimens were prioritised for testing [3, 21]. Syndromic surveillance data indeed suggest that ED attendances for GI attendances returned to normal levels more rapidly than GP in- and out-of-hours attendances, despite lower than usual ED attendances being observed over the 2020/21 and 2021/22 winter periods. This may reflect changes to health-seeking behaviour or may indicate changes to provision or issues with healthcare access. Many syndromic indicators, such as those for non-infectious gastrointestinal conditions, rapidly returned to baseline levels towards the end of the first UK lockdown, while gastrointestinal infections showed more modest increases [3]. NHS 111 call data and Google Trends are less likely to be impacted by healthcare capacity issues or health system changes. Both systems showed similar trends to those observed in other national surveillance systems, with reductions in activity observed until the lifting of control measures in the summer of 2021.

It is perhaps unsurprising that a true decrease in GI pathogen activity is likely to have occurred in England as many of the control measures implemented during the COVID-19 response are similar to those that would be implemented in response to a GI outbreak. Indeed, evidence suggests that NPIs implemented in the response were associated with reductions in other pathogens, such as influenza and respiratory syncytial virus [24-27]. Messaging around hand hygiene and physical distancing were consistent across the pandemic period with good adherence reported [28-29], and this has likely reduced person-to-person transmission of pathogens. The closure of schools and other settings has likely also led to a reduction in person-to-person transmission, while the closure of food premises and restrictions on mass gatherings and catered events have also probably reduced the risks associated with foodborne illness. Indeed, two peaks in activity were observed in the summer of 2020, corresponding to the reopening of restaurants to dine-in customers and England’s ‘Eat Out to Help Out’ scheme, respectively [30]. These findings are good evidence for the effectiveness of simple infection control measures such as hand washing and should strengthen the rationale for continuing public health campaigns aimed at reducing the spread of infectious diseases.

This study was strengthened by the triangulation of data from several national and regional-based surveillance systems; using this approach, we could determine that the trends observed were consistent across all indicators and show the importance of multiple surveillance systems to allow for comparative analysis across multiple indicators. However, this study is not comprehensive and there are other examples of operational surveillance systems, which have not been included in this work due to their limitations [3]. An additional limitation of this study is that negative laboratory test results are not captured by the SGSS laboratory surveillance system; therefore, it was not possible to determine to what degree the changes were due to changes in testing – we were also unable to calculate the positivity rate to assess whether only severe cases were being tested. As this study uses an ecological design, control measures implemented during the COVID-19 response cannot be proven to be the sole cause of changes in GI pathogen activity. Furthermore, the study does not take into account the geographical variation introduced by local or regional restriction measures, such as the three-tier system implemented between October 2020 and January 2021 whereby local authorities had different levels of restrictions [31], which would help to strengthen the relationship between control measures and GI pathogen activity.

This work is limited to describing trends in GI infections, and while it does not directly look at the drivers of this change, it does provide an indirect assessment. There is therefore scope for further research into the trends observed, for example, by performing in-depth pathogen-specific analyses to look for potential drivers such as socioeconomic factors and reduced foreign travel, which may have resulted in the trends observed.

## Supporting information

Love et al. supplementary material Love et al. supplementary material

## Data Availability

No additional data are available.

## Supplementary material

For supplementary material accompanying this paper visit http://doi.org/10.1017/S095026882300136X. click here to view supplementary material
