# Supplementary material for: Understanding the impact of the COVID-19 pandemic response on GI infection surveillance trends in England, January 2020–April 2022
Source: Epidemiol Infect. 2023 Aug 25;151:e147. doi: 10.1017/S095026882300136X (PMC10540168; doi:10.1017/S095026882300136X)
Supplement: Love et al. supplementary material [file S095026882300136Xsup001.docx]

**Supplementary Table 1 – Pandemic phases indicating key dates and policy measures implemented**

| **Phase** | **ISO week and year - start of phase** | **Week beginning date** | **Phase description** | **Key dates (Week beginning)** |
| --- | --- | --- | --- | --- |
| 1 | 1 (2020) | 30/12/2019 | Pre-outbreak period |  |
| 2 | 5 (2020) | 27/01/2020 | Advising of hygiene and social distancing measures prior to the first national lockdown | 27/01/2020 – First UK cases confirmed  09/03/2020 - Move from contain to delay, shielding announced  16/03/2020 - Avoid visiting social venues. Schools shut with exceptions, pubs and restaurants close. Working from home advised and furlough scheme announced. |
| 3 | 13 (2020) | 23/03/2020 | First national lockdown | 23/03/2020 - UK-wide partial lockdown announced  20/04/2020 - Key workers eligible for testing  04/05/2020 - Announcement on easing of measures  18/05/2020 – General public eligible for testing  25/05/2020 – NHS Test and Trace contacting tracing service goes live |
| 4 | 23 (2020) | 01/06/2020 | Gradual easing of restriction measures | 01/06/2020 – Phased reopening of schools commences  15/06/2020 – non-essential shops reopen  22/06/2020 - Relaxation of restrictions and social distancing announced  29/06/2020 – Pubs and restaurants reopen  03/08/2020 – Eat out to help out begins  10/08/2020 – Lockdown restrictions further eased. Entertainment venues reopen. |
| 5 | 38 (2020) | 14/09/2020 | Reimplementation of restriction measures | 14/09/2020 – Rule of 6 brought into force. Gatherings above 6 people banned.  21/09/2020 – Return of working from home. 10pm curfew on hospitality sector.  12/10/2020 – 3 tier system starts in England, with different restrictions in different areas |
| 6 | 44 (2020) | 26/10/2020 | Second and third national lockdowns | 26/10/2020 – second national lockdown announced  02/11/2020 – second national lockdown comes into force  23/11/2020 - 3 households able to meet during a 5-day Christmas period  30/11/2020 - Second lockdown ends, return to 3 tier restrictions  14/12/2020 - Tougher restrictions come into effect for SE and London (Tier 4). 21/12/2020 – More areas move into Tier 4  04/01/2021 – Third national lockdown |
| 7 | 10 (2021) | 08/03/2021 | Gradual easing of restriction measures | 08/03/2021 - Schools reopen. Recreation outdoors allowed between 2 people. Stay at home order remains in place.  29/03/2021 - Outdoor gatherings of 6 people or 2 households allowed. Outdoor sports facilities reopen. Stay at home order ends but people encouraged to stay local.  12/04/2021 - non-essential retail and public buildings reopen, outdoor leisure and entertainment venues reopen, self-contained holiday accommodation opens. Wider social contact rules remain with no indoor mixing between households allowed.  17/05/2021 - Limit of 30 people allowed to mix outdoors. Rule of 6 or two households allowed indoor social gatherings, indoor venues reopen including entertainment venues. Up to 10,000 spectators allowed to attend large outdoor venues such as football stadiums. |
| 8 | 29 (2021) | 19/07/2021 | Removal of legal limits on social contact and reopening of all closed sectors | 19/07/2021 - Most legal limits on social contact removed and final closed sectors reopen e.g., nightclubs  13/09/2021 - Plan B unveiled for winter if NHS comes under 'unsustainable pressure' |
| 9 | 49 (2021) | 06/12/2021 | Reintroduction of ‘Plan B’ measures to limit the spread of the emerging Omicron variant | 06/12/2021 - PM announces a move to Plan B following the spread of omicron variant. Lateral flow testing recommended when in high-risk settings or in contact with people not normally in contact with.  10/12/2021 - Face masks compulsory in most public venues.  13/12/2021 – Working from home advised  15/12/2021 – Proof of vaccination or negative lateral flow test required when entering venues where large crowds gather. |
| 10 | 9 (2022) | 28/02/2022 | Removal of all restrictions as part of the ‘Living with COVID-19’ strategy |  |

**Supplementary Figure 1 – Pandemic phases indicating average daily stringency index and average daily stringency index for each phase**


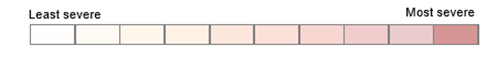


Most stringent

Least stringent


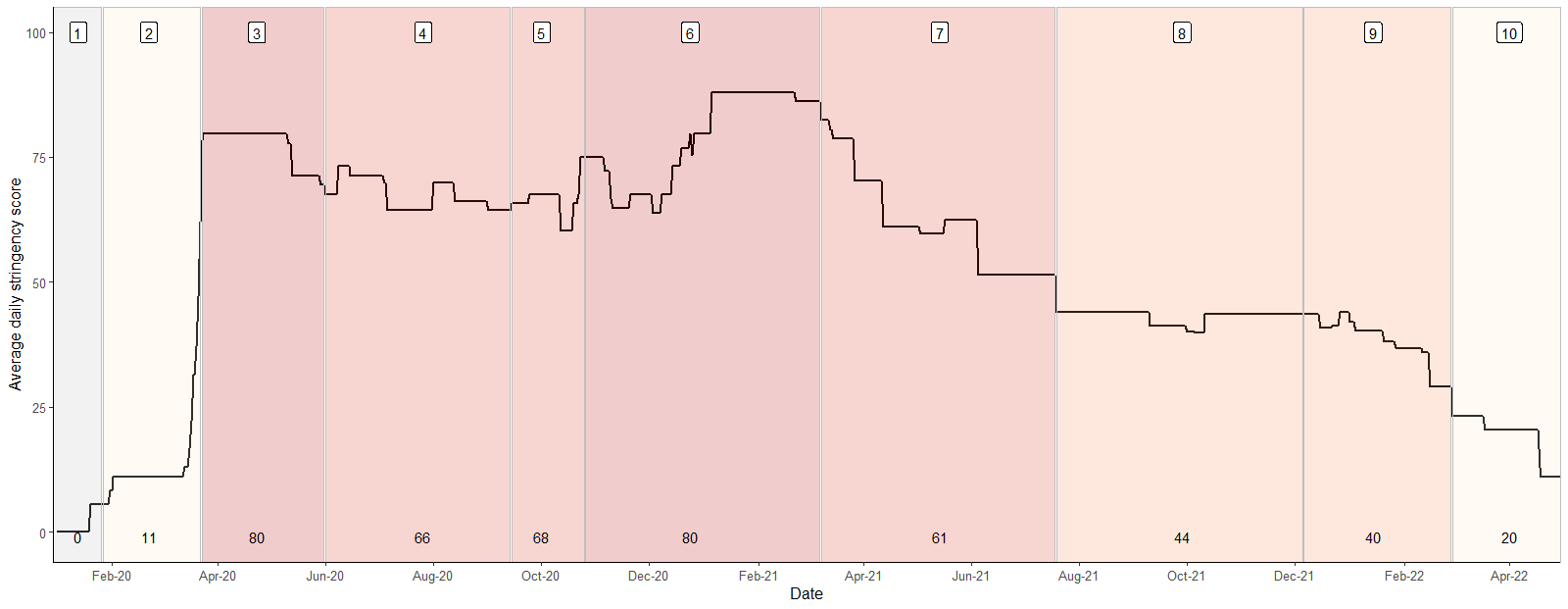


*Pandemic phases are assigned based on control measures implemented during the pandemic response, using the Oxford Stringency Index which indicates the severity of government restrictions in England [13] from least severe measures to most severe measures. A daily stringency index was calculated based on the mean score of nine metrics: school closures; workplace closures; cancellation of public events; restrictions on public gatherings; closures of public transport; stay-at-home requirements; public information campaigns; restrictions on internal movements; and international travel control, each taking a value between 0 and 100, with 100 being the strictest response. This score was converted to deciles, as displayed in the bar at the top of the figure. Grey shaded area indicates no restriction measures in place.*
